# Genome-wide analysis and identification of the *PEBP* genes of *Brassica juncea* var. *Tumida*

**DOI:** 10.1186/s12864-022-08767-3

**Published:** 2022-07-23

**Authors:** Jing He, Linxin Gu, Quanqin Tan, Yu Wang, Fanfan Hui, Xiaohong He, Pingan Chang, Daping Gong, Quan Sun

**Affiliations:** 1 College of Bioinformation, Chongqing Key Laboratory of Big Data for Bio Intelligence, Chongqing University of Posts and Telecommunications, Chongqing, China; 2 Tobacco Research Institute of Chinese Academy of Agricultural Sciences, Qingdao, China

**Keywords:** *PEBP*, Tumorous stem mustard, Gene expression, Flowering, Bolting, *Brassica juncea* var. *Tumida*

## Abstract

**Background:**

Phosphatidylethanolamine-binding protein (PEBP) is widely present in animals, plants, and microorganisms. Plant *PEBP* genes are mainly involved in flowering transition and nutritional growth. These genes have been studied in several plants; however, to the best of our knowledge, no studies have explored them in *Brassica juncea* var. *tumida*. This study identified and characterized the entire *PEBP* gene family of *Brassica juncea* var. *tumida.*

**Results:**

A total of 21 *PEBP* genes were identified from *Brassica juncea* var. *tumida*. Through phylogenetic analysis, the 21 corresponding proteins were classified into the following four clusters: TERMINAL FLOWER 1 (TFL1)-like proteins (*n* = 8), MOTHER OF FT AND TFL1 (MFT)-like proteins (*n* = 5), FLOWERING LOCUS T (FT)*-*like proteins (*n* = 6), and ybhB-like proteins (*n* = 2). A total of 18 genes contained four exons and had similar gene structures in each subfamily except *BjMFT1*, *BjPYBHB1*, and *Arabidopsis thaliana CENTRORADIALIS* homolog of *Brassica juncea* var. *tumida (BjATC1)*. In the analysis of conserved motif composition, the *BjPEBP* genes exhibited similar characteristics, except for *BjFT3*, *BjMFT1*, *BjPYBHB1*, *BjPYBHB2*, and *BjATC1*. The *BjPEBP* promoter includes multiple cis-acting elements such as the G-box and I-box elements that respond to light, ABRE and GARE-motif elements that respond to hormones, and MBSI and CAT-box elements that are associated with plant growth and development. Analysis of RNA-Seq data revealed that the expression of a few *BjPEBP* genes may be associated with the development of a tumorous stem. The results of qRT–PCR showed that *BjTFL1* and *BjPYBHB1* were highly expressed in the flower tissue, *BjFT1* and *BjATC1* were mainly expressed in the root, and *BjMFT4* were highly detected in the stem. The results of yeast two-hybrid screening suggested that BjFT interacts with Bj14-3-3. These results indicate that *BjFT* is involved in flowering regulation.

**Conclusions:**

To the best of our knowledge, this study is the first to perform a genome-wide analysis of *PEBP* genes family in *Brassica juncea* var. *tumida*. The findings of this study may help improve the yield and molecular breeding of *Brassica juncea* var. *tumida*.

**Supplementary Information:**

The online version contains supplementary material available at 10.1186/s12864-022-08767-3.

## Background

Tumorous stem mustard (*Brassica juncea* var. *tumida*) is a dicotyledonous plant belonging to *Brassicaceae* family of cruciferous crops [1]. The genus *Brassica* mainly includes three diploid species (*Brassica rapa* [AA]), *Brassica nigra* [BB], and *Brassica oleracea* [CC]) and three allopolyploid species (*Brassica napus L.* [AACC], *Brassica juncea* [AABB], and *Brassica carinata* [BBCC]). *Brassica juncea* is produced through hybridization between the diploid ancestors of *Brassica rapa* and *Brassica nigra* [2]. The evolutionary relationships among these *Brassica* species can be described using the well-known “triangle of U” model. Tumorous stem mustard is a major vegetable crop that has high economic value because of its primary use as a fresh vegetable or a raw material for Fuling mustard [3–5]. Tumorous stem mustard crops are majorly distributed in Chongqing, Zhejiang, Sichuan, Hunan, and Hubei in the Yangtze River basin, East China. The growth of *Brassica juncea* var. *tumida* involves four stages: germination, seedling, stem swelling, and flowering. However, owing to the influence of variety, photoperiod, and cultivation conditions, this crop may transit early from vegetative to reproductive growth. These factors often lead to early flowering and bolting, which reduces crop yield.

Phosphatidylethanolamine-binding protein (PEBP) is a class of evolutionarily conserved proteins that are widely present in plants, animals, microorganisms [6–8]. It plays an important role in regulating floral transition and seed germination [9–11]. Six *PEBP* genes have been reported in the model plant *Arabidopsis thaliana*: *FLOWERING LOCUS T* (*FT*), *TWIN SISTER OF FT* (*TSF*), *TERMINAL FLOWER 1* (*TFL1*), *BROTHER OF FT AND TFL1* (*BFT*), *MOTHER OF FT AND TFL1* (*MFT*), and *Arabidopsis thaliana CENTRORADIALIS* (*ATC*) [12, 13]. They were classified into three subfamilies: *FT*-like, *TFL1*-like, and *MFT*-like subfamilies [10]. Recently, a new member of this gene family, AT5G01300 (*PYBHB*), was detected in *Arabidopsis thaliana* by Sheng et al. [14]. They classified it into the fourth subfamily called the *ybhB*-like subfamily [15]. Thus far, a total of seven *Arabidopsis PEBP* genes have been identified. *Arabidopsis FT*, *TSF*, and *MFT* promote flowering and *TFL1*, *ATC*, and *BFT* repress it [16–19]. *FT* belongs to the *FT-*like subfamily; it is a florigen encoding gene [18, 19]. Recent studies have identified several regulatory pathways associated with flowering: photoperiod, temperature-sensitive, vernalization, autonomous, hormone, and age pathways [20–22]. By integrating signals sensed by the photoperiodic, vernalization, and autonomous pathways, FT protein plays a major role in the photoperiodic pathway as a flowering regulation integrator [23], downstreaming flowering development *CONSTANS* (*CO*). Under prolonged daylight conditions, CO proteins induce the expression of *FT* genes [24]. FT protein is transferred from the leaves to the shoot apical meristem, and it then binds to FD protein [24]. These complexes induce the expression of the following genes: *SUPPRESSOR OF OVEREXPRESSION OF CONSTANS1*, *FRUITFUL*, and *APETALA1 (AP1)* [25, 26]. *TFL1* belongs to the *TFL1*-like subfamily. Unlike *FT*, *TFL1* inhibits the plant’s transition from inflorescence meristem to floral meristem, thus delaying flowering time [27]. *TFL1* functions in infinite inflorescence branching species by maintaining infinite inflorescence growth and in limited inflorescence branching species by flowering transition and inflorescence structure maintenance. In *Arabidopsis* sp., *TFL1* regulates the meristem genes *LEAFY (LFY)* and *AP1* to control the plant’s morphological structure [28, 29]. *MFT* belongs to the *MFT*-like subfamily and is the ancestor of *FT* and *TFL1*. Overexpressed *AtMFT* leads to early flowering, but this exhibits a weak activity in the promotion of flowering. *MFT* is expressed in seed in *Arabidopsis thaliana*, and regular seed germination through the abscisic acid (ABA) and gibberellic acid (GA) signaling pathways [30].

The *PEBP* family has been identified in various plants such as *Moso Bamboo* (gene number [n] = 6) [31], *Oryza sativa* (*n* = 19) [11], *Gossypium hirsutum* (*n* = 8) [32], common wheat (*n* = 76, 38, 16, and 22) [33], *Glycine max* (*n* = 27) [34], *Vitis cinifera* (*n* = 5) [35], *Rosaceae* tree species (*n* = 56) [36], rice (*n* = 19) [11], and corn (*n* = 25) [37].

Because the entire *Brassica juncea* var. *tumida* genome has been sequenced [2], a genome-wide analysis of *PEBP* genes was performed for the first time in this study. The phylogenetic relationship, gene structure, protein motif, chromosome location, and expression profile of a total of 21 identified *BjPEBP* genes in different tissues were analyzed. The results may provide valuable information for classifying *BjPEBP* genes and lay the foundation for exploring the molecular mechanisms underlying stem swelling and flowering orchestrated by *PEBP* genes in *Brassica juncea* var. *tumida.*

## Results

### Identification of the *PEBP* family members of *Brassica juncea* var*. tumida*

In this study, a total of 21 genes were identified in *Brassica juncea* var. *tumida* using the protein families database (Pfam), National Center for Biotechnology Information (NCBI), Conserved Domains Database (CDD), and Simple Modular Architecture Research Tool (SMART) database. These 21 *BjPEBP* genes were found to possess the typical *PEBP* domain (PF01161) and were named in reference to *AtPEBPs* (Table 1). These *BjPEBP* genes possess only one *PEBP* domain, except *BjATC1* that possesses two *PEBP* domains. The number of coding amino acids ranges from 135 to 281; *BjMFT1* and *BjATC1-1* are 135-aa and 281-aa long, whereas the others are approximately 175-aa long. The isoelectric point ranged from 5.34 to 9.69. These *BjPEBP* proteins were mainly subcellularly located on the cytoplasm (Table 1).

**Table 1:** The *PEBP* genes family members in *Brassica juncea* var. *tumida*

Gene_id	Gene_name	chr	Start-end	Sence+/antisence-	Pfam domain	Protein(aa)	MW	PI	Subcellular location	At homolog
BjuB048175	BjFT1	B02	33,175,908–33,178,027	+	130	175	19810.43	7.75	Chlo、cyto、nucl、mito、plas	AtFT
BjuO006613	BjFT2	Contig429	24,278–26,797	+	132	175	19810.43	7.75	Chlo、cyto、nucl、plas	AtFT
BjuB043970	BjFT3	B03	27,650,574–27,653,574	-	116	170	19197.79	8.68	Cyto、chlo、extr、nucl	AtFT
BjuA029389	BjTSF1	A07	18,288,301–18,293,021	-	134	176	19945.67	7.72	Chlo、cyto、extr	AtTSF
BjuB020451	BjBFT1	B06	6,684,085–6,684,954	+	137	177	20061.06	9.45	cyto、nucl、plas、cysk	AtBFT
BjuA022875	BjBFT2	A06	17,261,662–17,262,565	+	137	177	20146.12	9.51	cyto、nucl、plas、cysk	AtBFT
BjuA022180	BjMFT1	A06	6,713,651–6,722,725	-	99	135	14790.04	6.82	cyto、extr	AtMFT
BjuA044292	BjMFT2	A09	50,665,724–50,667,446	+	132	173	19070.04	8.79	cyto、extr	AtMFT
BjuB032383	BjMFT3	B03	15,228,277–15,229,987	-	132	173	19070.04	8.79	cyto、extr	AtMFT
BjuB022680	BjMFT4	B04	21,491,623–21,493,549	-	131	173	19062.04	8.76	cyto、extr	AtMFT
BjuB029247	BjMFT5	B04	19,746,538–19,748,461	-	131	173	19062.04	8.76	cyto、extr	AtMFT
BjuB038969	BjTFL1	B08	862,896–863,943	-	135	177	20154.29	9.69	cyto、extr	AtTFL1
BjuA008949	BjTFL2	A03	804,140 -805,199	-	135	177	20111.22	9.56	cyto、extr	AtTFL1
BjuB012624	BjTFL3	B05	18,474,225–18,475,277	+	135	179	20363.36	8.79	cyto、extr	AtTFL1
BjuB048699	BjTFL4	B02	52,307,789–52,308,864	+	137	178	20269.2	9	cyto、extr、pero	AtTFL1
BjuA040052	BjTFL5	A10	18,936,879–18,937,941	+	137	178	20401.45	9.51	cyto、extr、plas	AtTFL1
BjuB004009	BjPYBHB1	B06	27,302,591–27,303,332	+	143	162	17794.28	5.34	cyto、extr、E.R	PYBHB
BjuA040212	BjPYBHB2	A10	19,806,740–19,808,567	-	142	314	33748.74	6.03	Chlo、mito	PYBHB
BjuA026251	BjATC1	A07	18,288,301–18,293,021	+	112/137	281	31492.97	9.51	cyto	AtATC
BjuB046109	BjATC2	B08	30,494,732–30,496,447	+	131	175	19877.74	7.85	cyto、nucl	AtATC
BjuB021008	BjATC3	B06	11,137,277–11,138,906	-	130	174	19804.64	6.58	Cyto	AtATC

*Chlo* Chloroplast, *cyto *cytoplasm, *extr *Cytoplasmic matrix, *nucl *nucleus, *pero *peroxisome, *plas *Plasma membrane, *E.R *Endoplasmic reticulum, *mito *Mitochondria

**Fig. 1:**
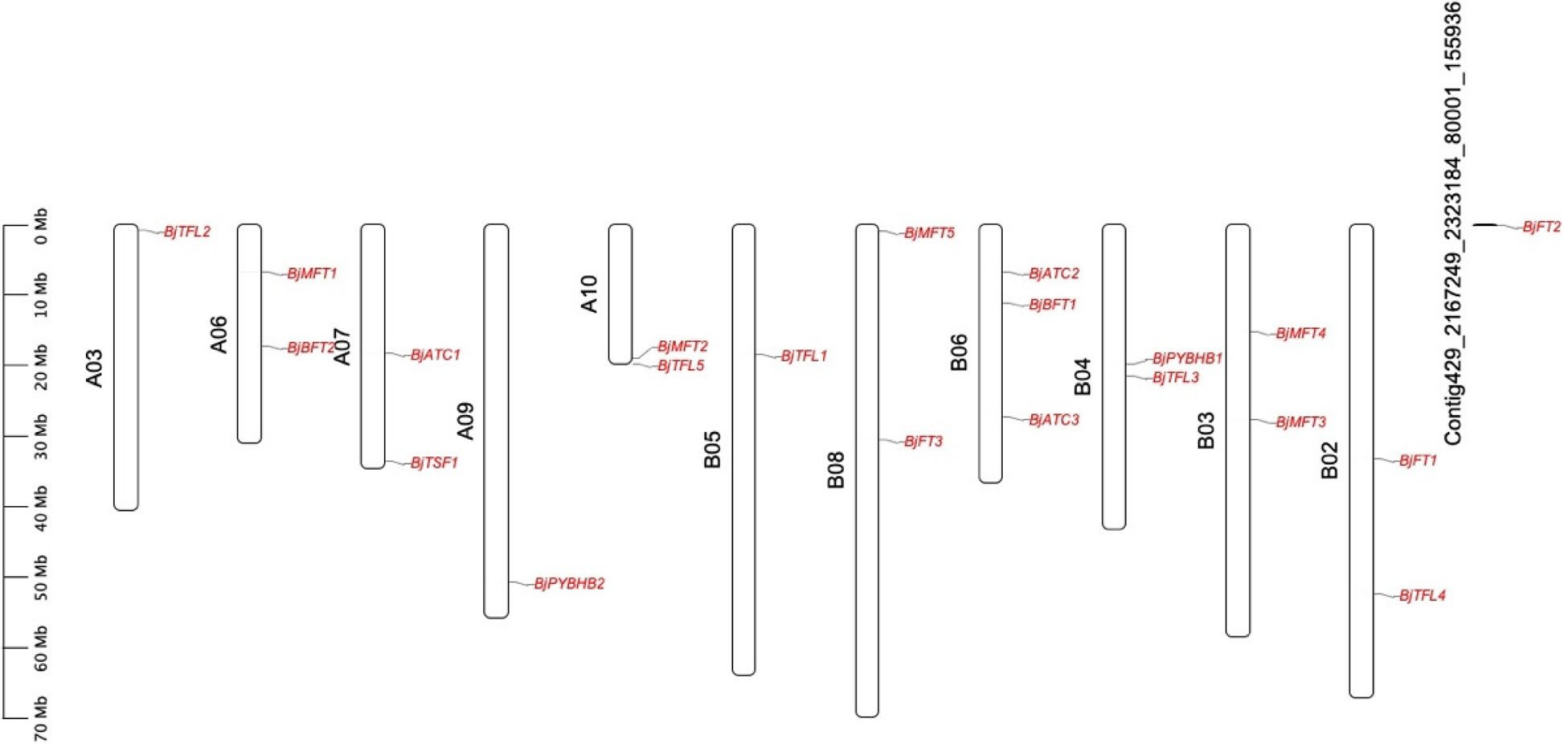
The gene locations of *BjPEBP*gene family. The chromosome name is at the top of each bar. The scale of the chromosome is in millions of bases (Mb)

Of the 21 genes, 20 were located on 11 chromosomes, except *BjFT2*, which was anchored in contig429. There was one *BjPEBP* gene each on chromosomes A03, A09, and B05; two *BjPEBP* genes each on chromosomes A06, A07, A10, B02, B03, B04, and B08; and three *BjPEBP* genes on chromosome B06 (Fig. 1).

### Construction of a molecular evolutionary tree of *PEBP* genes

**Fig. 2:**
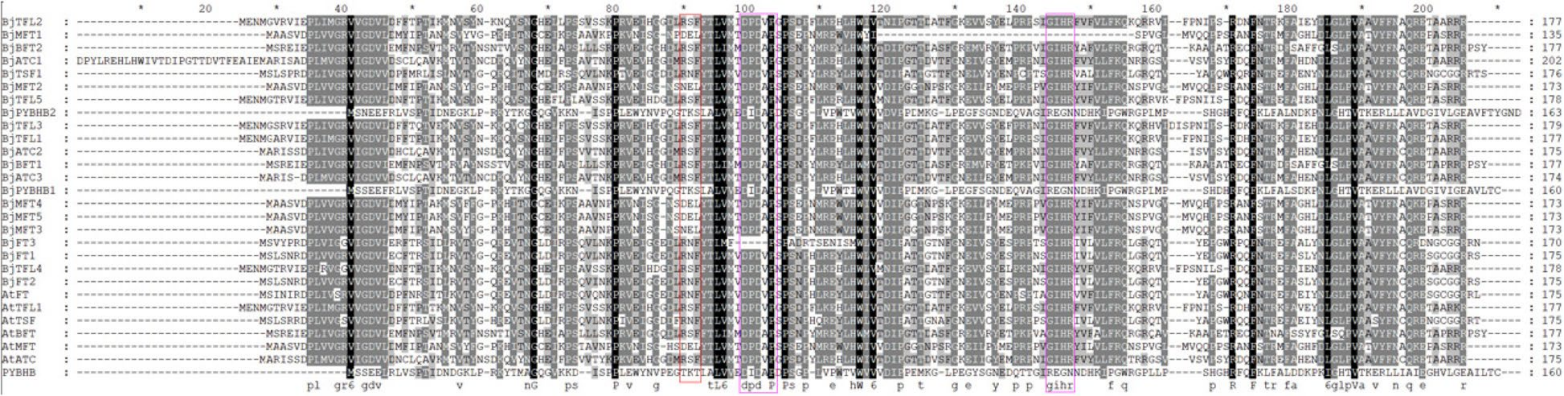
Sequence alignment of 28 PEBP proteins of *Brassica juncea* var. *tumida* and *Arabidopsis.* The sequences were aligned using ClustalW. The conserved protein motif 14-3-3 interaction interface and anion-binding site are rectangle with the color of red and pink, respectively. The rest is not shown

**Fig. 3:**
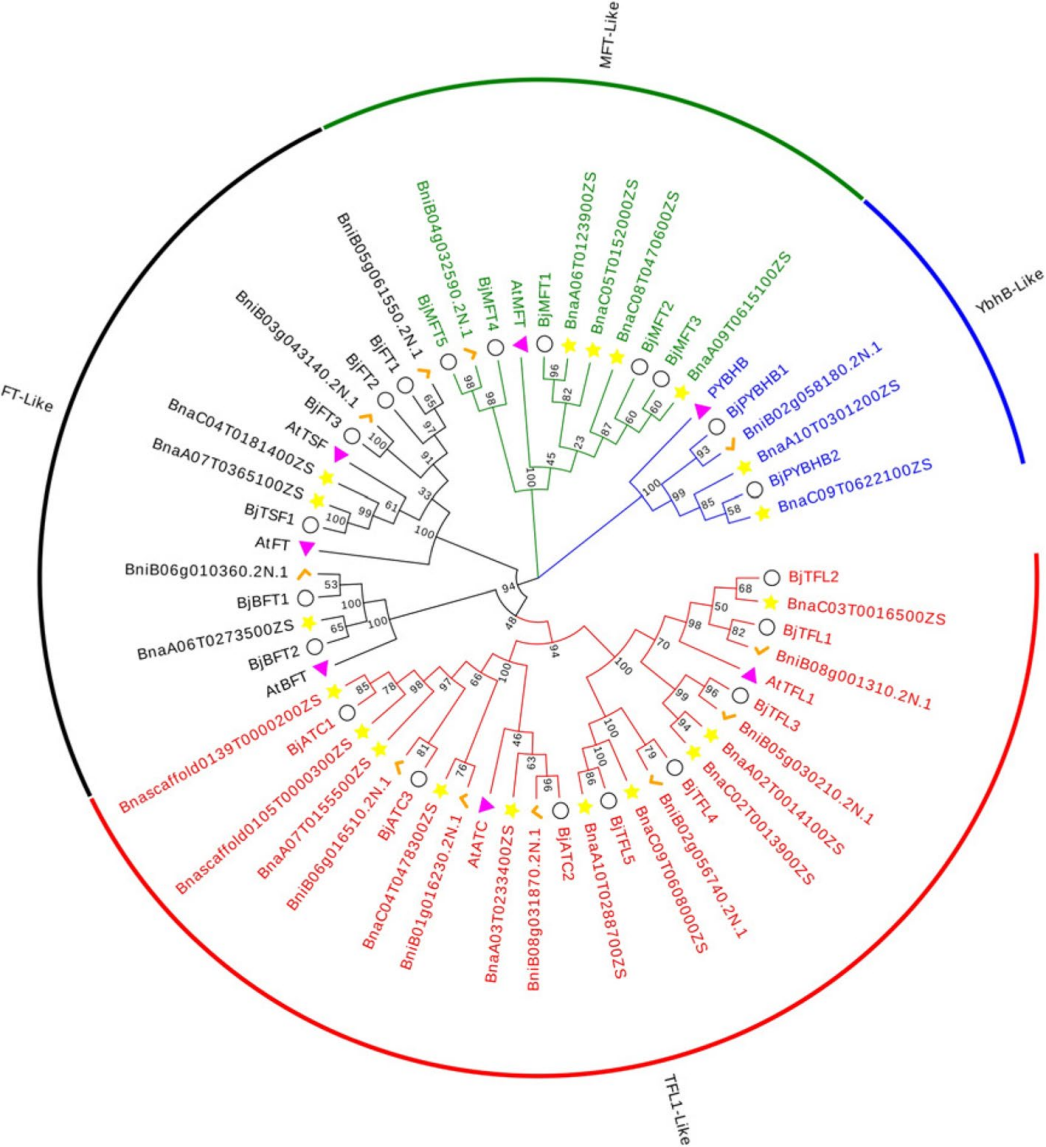
Molecular evolutionary analysis of PEBP proteins from *Arabidopsis*, *Brassica juncea* var. *tumida*, *Brassica napus L.* and *Brassica nigra*. Star: the *PEBP* genes of *Brassica napus L.* Triangle: the *PEBP* genes of *Arabidopsis.* Check: the *PEBP* genes of *Brassica nigra.* The evolutionary history was inferred using the Neighbor-Joining method. The optimal tree is shown. The percentage of replicate trees in which the associated taxa clustered together in the bootstrap test (1000 replicates) are shown next to the branches. The evolutionary distances were computed using the Poisson correction method and are in the units of the number of amino acid substitutions per site. This analysis involved 58 amino acid sequences. All ambiguous positions were removed for each sequence pair (pairwise deletion option). There were a total of 314 positions in the final dataset. Evolutionary analyses were conducted in MEGA X

To further elucidate the evolutionary relationship among the members of the *PEBP* gene family, an unrooted molecular evolutionary tree was constructed using the neighbor-joining (NJ) method; the 21 identified *BjPEBPs* of *Brassica juncea* var. *tumida* and 7 *AtPEBPs* of *Arabidopsis* were analyzed. PEBP proteins were subjected to multiple sequence alignment via ClustalW; the results showed that most proteins possess an interaction site for 14-3-3 protein (RXF motif), and all proteins possess an anion-binding site (GIHR and DPDxP motif) (Fig. 2). The evolutionary tree constructed using the NJ method with *Arabidopsis*, *Brassica juncea* var. *tumida*, *Brassica napus L.*, and *Brassica nigra* indicated that the genes could be divided into four subfamilies (Fig. 3): *FT-*like, *TFL1*-like, *MFT*-like, and *ybhB*-like subfamilies. In *Brassica juncea* var. *tumida*, the *MFT*-like subfamily comprises five members: *BjMFT1*, *BjMFT2*, *BjMFT3*, *BjMFT4*, and *BjMFT5.* The *TFL1*-like subfamily comprises eight members: *BjTFL1*, *BjTFL2*, *BjTFL3*, *BjTFL4*, *BjTFL5*, *BjATC1*, *BjATC2*, and *BjATC3.* Furthermore, the *FT-*like subfamily comprises six members: *BjBFT1*, *BjBFT2*, *BjFT1*, *BjFT2*, *BjFT3*, and *BjTSF1.* Finally, the *ybhB*-like subfamily comprises two members: *BjPYBHB1* and *BjPYBHB2*.

### Analysis of the gene structure and conserved motifs of *BjPEBPs*

**Fig. 4:**
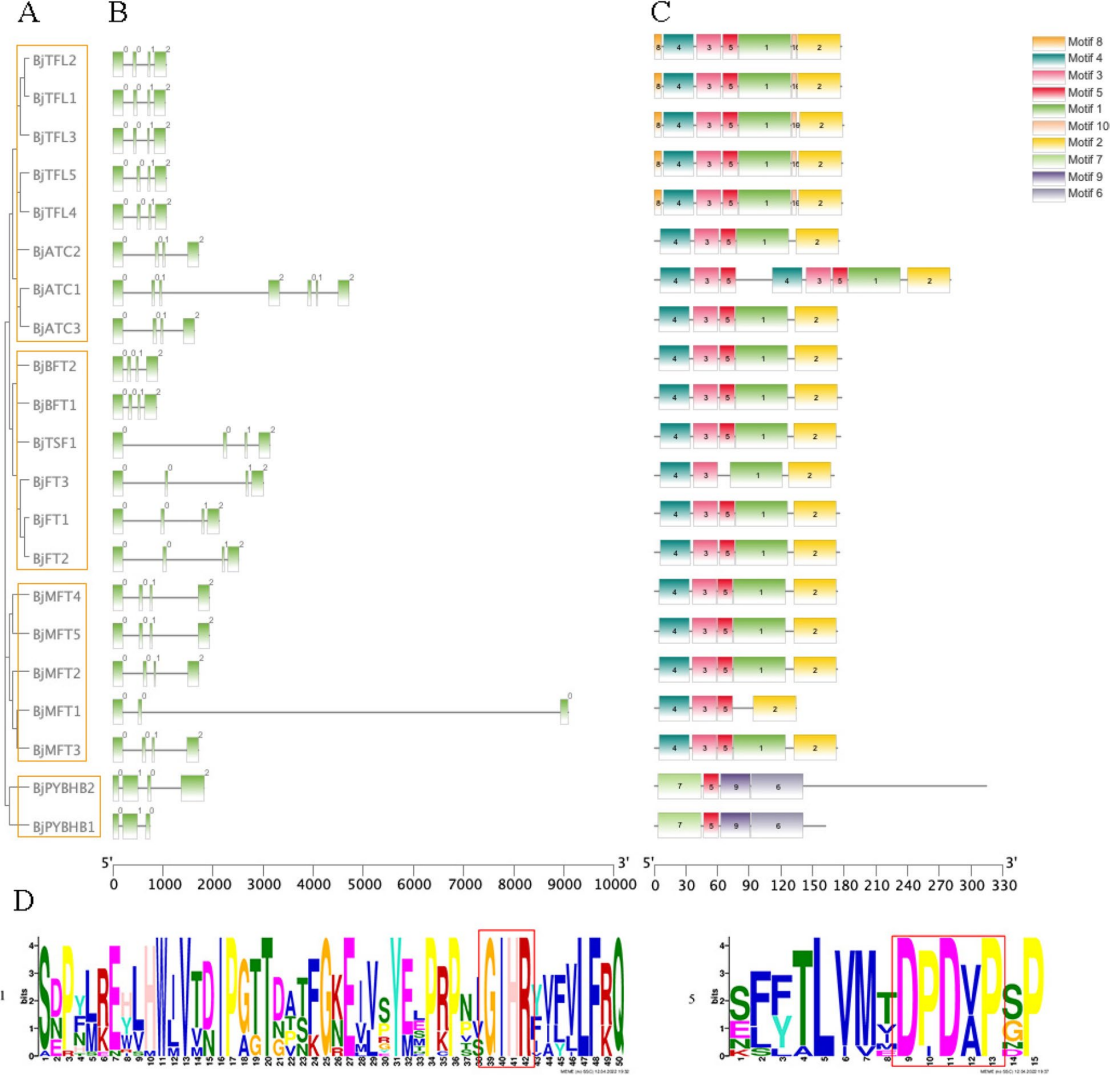
Genomic structure and motif composition of *BjPEBPs.* (**A**) The phylogenetic tree of BjPEBP proteins. (**B**) Genomic structure of *BjPEBP* genes family members in *Brassica juncea* var. *tumida*. Exons and introns are indicated with green boxes and black lines. (**C**) The conserved motifs in *Brassica juncea* var. *tumida* PEBP proteins identified using MEME online website. Each motif is indicated with a special color. (**D**) Two major motif logo of *BjPEBPs*. The character sequence of *BjPEBPs* motif

In this study, *BjPEBP* genes could be divided into four categories (Fig. 4 A). The gene structure indicated that most *BjPEBP* genes have four exons, except *BjATC1*, which contains seven exons, and *BjPYBHB1* and *BjMFT1*, which contain three exons each. The sizes of exons and introns in the same cluster genes showed high similarity (Fig. 4B). The conserved motifs present in the 21 BjPEBP proteins were identified (Fig. 4 C). In total, 10 motifs were identified: motifs 1–10. The *BjPEBP* genes contain motif1, motif2, motif3, motif4, and motif5, except *BjMFT1*, *BjPYBHB1*, and *BjPYBHB2*. The character sequence of the *BjPEBPs* motif helps identify the motif that is conserved and can bind an anion. (Fig. 4D).

### Analysis of promoter cis-acting elements of *BjPEBP* genes

**Fig. 5:**
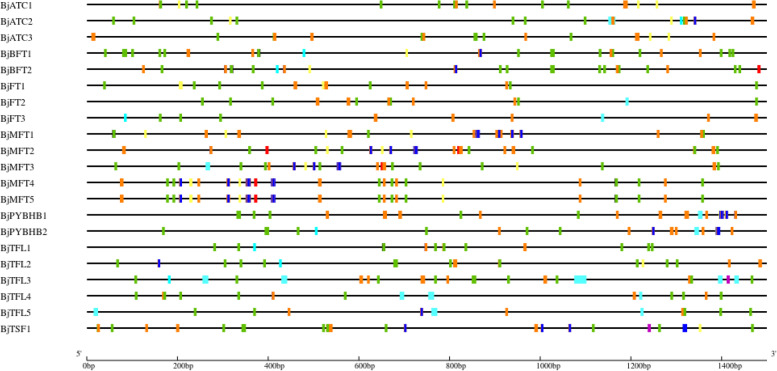
Cis-acting elements on promoters of *BjPEBP* genes. Orange mean Light response elements. Green showed involved in biotic or abiotic stress response components. Cyan showed elements related to growth and development. Blue showed the element of ABRE. Black showed the element of GARE-motif. Red showed the element of P-box. Purple showed the element of TATC-box. Other hormome response elements were showed yellow

The promoter cis-acting elements of a gene are associated with its expression and function. In this study, multiple promoter cis-acting elements in were observed in *BjPEBP* promoters. There are four primary types of cis-acting elements (Table 2; Fig. 5): light-responsive, hormone-responsive, biotic or abiotic stress response, and growth and development–related elements.

**Table 2:** The information of *BjPEBP* genes promotor cis-acting element

function	name
response to hormomes element	ABRE、GARE-motif、P-box、CGTCA-motif、TGACG-motif、AuxRR-core、TATC-box
Light response elements	AE-box、G-box、G-Box、GA-motif、GT1-motif、Gap-box、I-box、LAMP-element、MRE、sp1、TCT-motif、chs-CMA2a
Involved in biotic or abiotic stress response components	MBS、ARE、LTR、GC-motif、TC-rich、MYB、MYC
elements related to growth and development	HD-zip、MBSI、MSA-like、circadian、GCN4-motif、CAT-box

The following hormone-responsive cis-acting elements were identified: ABRE, MeJA response elements (CGTCA-motif and TGACG-motif), GARE-motif, p-box, and growth-hormone response element (AuxRR-core); they are mostly present in the members of the *MFT*-like and *TFL1*-like subfamilies. The following 11 light-responsive cis-acting elements were also identified: AE-box, G-box, GA-motif, GT1-motif, Gap-box, I-box, LAMP-element, MRE, sp1, TCT-motif, and chs-CMA2a. Furthermore, the following six growth and development–related elements were detected: HD-zip, MBSI, MSA-like, circadian, GCN4-motif, and CAT-box. The biotic or abiotic stress response elements identified were as follows: MBS, ARE, LTR, GC-motif, TC-rich, MYB, and MYC. Nearly all genes contain the aforementioned regulatory elements, except for *BjATC1*, *BjATC3*, *BjFT1*, *BjMFT1*, *BjMFT2*, *BjMFT3*, *BjMFT4*, *BjMFT5*, *BjPYBHB1*, and *BjTSF1*. These genes do not contain any growth and development–related elements. In *BjTFL1*-like genes, except *BjTFL2*, *BjATC1–3* contain hormone-responsive cis-acting element, but the other genes do not contain this regulatory cis-acting element in their promoters. Among these four types of elements, the light-responsive and biotic or abiotic stress response elements were the most diverse and numerous. The abundant information on cis-acting elements suggest that this gene family is be involved in various regulatory mechanisms and play an important role in the stress response as well as growth and development of *Brassica juncea* var. *tumida*.

### Expression of *BjPEBP*genes

**Fig. 6:**
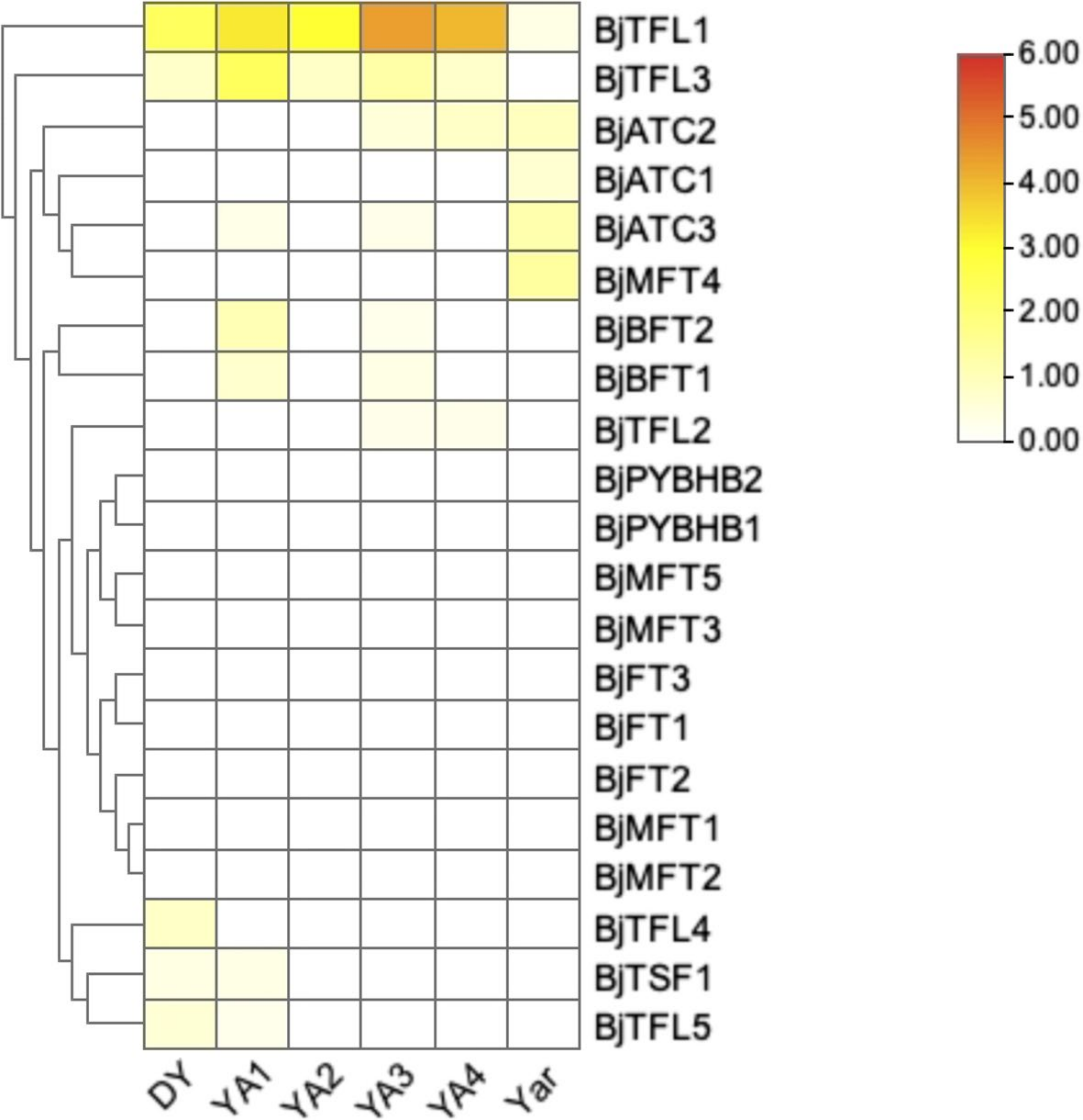
Expression patterns of *BjPEBP* genes in different development stages. DY, Dayejie stems were collected 22 weeks after seeding (daye3bianzhong); YA1-4, The stems of Yongan were collected 18, 20, 22, and 25 weeks after seeding; YAr, The mix roots samples of 18 and 22 weeks after seeding. The expression levels are represented by the color bar

Based on RNA-Seq data collected in a previous study, the expression patterns of *BjPEBP* genes in different tissues were analyzed [38]. The expression of *BjTFL1*, *BjTFL2*, *BjATC2*, *BjATC1*, *BjATC3*, *BjMFT4*, *BjBFT2*, *BjBFT1*, *BjTFL4*, *BjTSF1*, and *BjTFL5* genes were detected in at least one type of tissue (Fig. 6). *BjTFL1* and *BjTFL3* belong to the *TFL1*-like subfamily, and their expression pattern was similar. They were expressed in almost all tissues, except for *BjTFL3*, which was not detected in the root. The expression of *BjTFL1* and *BjTFL3* was increased and then decreased from YA1 to YA4. *BjATC2* expression was detected in YA3, YA4, and YAr, whereas *BjATC1* expression was detected in only YAr. *BjMFT4* expression was similar to that of *BjATC1*. The expression of *BjBFT1*, *BjBFT2*, and *BjATC3* was detected in YA1 and YA3. *BjATC3* expression was also detected in YAr. *BjTFL2* expression was noted to be weak in YA3 and YA4. *BjTFL4*, *BjTFL5*, and *BjTSF1* showed low expression in Dayejie (DY), YA1, and YA2. The expression of other genes in these tissues remained undetected.

**Fig. 7:**
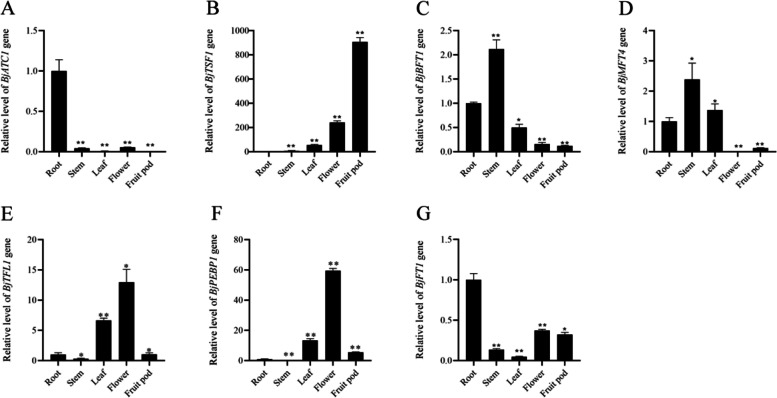
Expression levels of seven *PEBP* genes in *Brassica juncea* var. *tumida* different tissues by qRT-PCR. Statistically significant differences between tissues are indicated using asterisks (*p < 0.05, **p < 0.001; independent t-test)

The expression of *BjATC1, BjTSF1, BjBFT1, BjMFT4, BjTFL1, BjPYBHB1, and BjFT1* in plant tissues was further detected via qRT–PCR. *BjATC1* exhibited weak expression in the tissues except the root (Fig. 7 A). *BjTSF1* showed high expression in the leaf, flower, and fruit pod, with the highest expression detected in the fruit pod (Fig. 7B). *BjBFT1* showed a higher expression in the stem, followed by that in the root and leaf; the lowest expression was detected in the flower and fruit pod (Fig. 7 C). *BjMFT4* and *BjBFT1* exhibited a similar expression pattern (Fig. 7D). Furthermore, *BjTFL1* and *BjPYBHB1* showed a similar expression pattern in the tissues. These genes exhibited high expression in the flower and leaf and weak expression in the fruit pod, root, and stem (Fig. 7E and F). *BjFT1* showed a higher expression in the root, flower, and fruit pod than in the stem and leaf (Fig. 7G).

### Subcellular localization of BjFT1

**Fig. 8:**
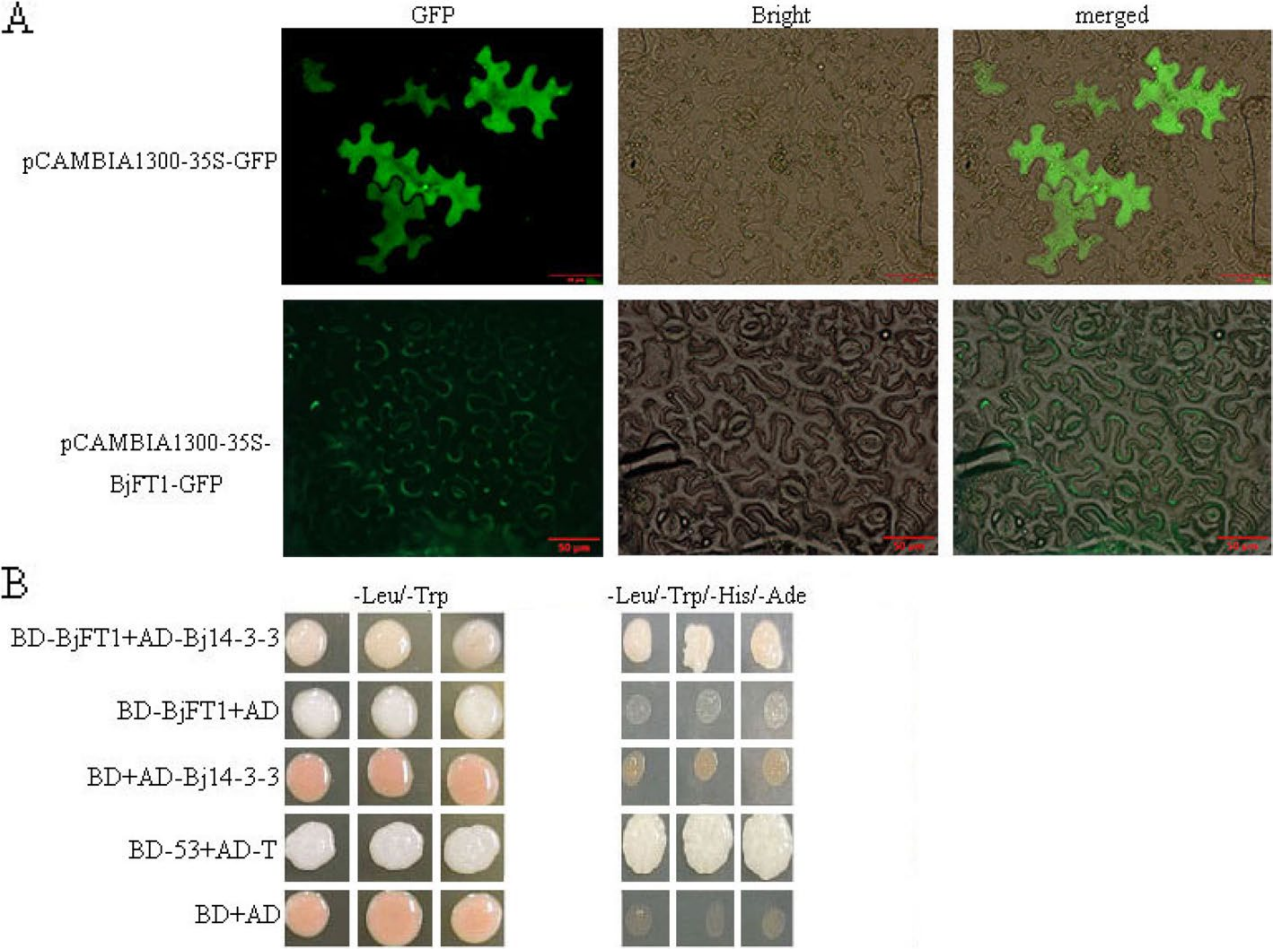
Subcellular localization and the interaction of BjFT1 protein. (**A**) Cells with only *GFP* reporter gene and *BjFT1* gene under fluorescence and white light. The scale bar is 50 μm. (**B**) Yeast two-hybrid assay of BjFT1-Bj14-3-3 interaction. The interaction of BjFT1 and Bj14-3-3 in yeast cells. BD-53 + AD-T and BD + AD as the positive and negative controls, respectively. The yeast co-transformed BD-BjFT1 + AD-Bj14-3-3, BD-BjFT1 + AD, BD + AD-Bj14-3-3 and the control groups grown on the SD-Leu-Trp medium, and then grown on the SD-Leu-Trp-His-Ade medium

The subcellular localization of a protein helps predict its functions. The BjFT1–GFP fusion protein was transiently expressed in tobacco leaves. The results of fluorescence analysis revealed that BjFT1–GFP is accumulated in the plasma membrane (Fig. 8 A).

### BjFT1 interacts with Bj14-3-3

Most members of the *PEBP* family possess interaction sites for the members of the 14-3-3 family proteins. 14-3-3 may interacts with FT/Hd3a in cytoplasm and then the FT/Hd3a-14-3-3 complex interacts with FD, which is called the florigen activation complex (FAC) [39]. In this study, one *PEBP* gene, *BjFT1*, and one *Bj14-3-3* gene were selected for to assess the interaction. The result showed that the experimental (pGBKT7::*BjFT1* and pGADT7::*Bj14-3-3*) and positive (pGBKT7-53 + pGADT7-T) groups grew well on the SD-Leu-Trp and SD- Leu-Trp-His-Ade media. Thus, BjFT1 and Bj14-3-3 appear to interact with each other (Fig. 8B).

## Discussion

Plant *PEBP* genes are associated with flowering and growth development. These were conserved in many plants. In *B. juncea* var. *tumida*, *B. napus L.*, and *B. nigra*, a total of 21, 19, and 11 *BjPEBP* genes were identified, respectively. A previous study identified *PEBP* genes in *Arabidopsis* sp. [14]. Therefore, as tetraploid plants, *Brassica juncea* var. *tumida* and *Brassica napus* L. possess nearly three times more *PEBP* family genes than *Arabidopsis* sp. The *BjPEBP* gene family comprises three *ATC*, five *TFL*, five *MFT*, two *BFT*, three *FT*, one *TSF*, and two *PYBHB* genes. A total of 11 *BjPEBP* genes were identified in *B. nigra* (BB), a number higher than that noted for *Arabidopsis* sp. *B. juncea* is a tetraploid derived from the hybridization of *B. rapa* (AA) and *B. nigra* (BB); the increase in the number of *BjPEBP* genes might have resulted before the formation of the tetraploid. *TFL*-like genes play an important role in nutritional growth and inflorescence meristem-specific growth maintenance [10]. Five *TFL*-like subfamily genes are present in *Brassica juncea* var. *tumida*, which may originate through a multifunctional differentiation of *TFL* genes during growth and development. Different *BjTFL* genes regulate specific pathways. In *Arabidopsis*, the expression of *BFT* gene was upregulated under ABA, drought, and osmotic stress conditions. *BFT* genes may play a regulatory role in flowering time and inflorescence structure under drought conditions [40]. In *Brassica juncea* var. *tumida*, two *BjBFT* genes that may be closely associated with flowering and stress response function and the domestication of this species were identified.

Regarding the structural composition of *PEBP*, all *PEBP* genes were found to have four exons and three introns, except *BjMFT1* and *BjPYBHB1*; this finding is consistent with that of Zhang et al. [36] who identified the *PEBP* gene family in nine *Rosaceae* trees species. The second and third exons of *BjPEBP* were noted to be short and the first and fourth exons were noted to be long; this finding is similar to that observed in *Jatropha curcas* [41]. The short motifs DPDxP (Asp-Pro-Asp-X-Pro) and GIHR (Gly-Ile-His-Arg) are highly conserved and represent the characteristic motifs of the *PEBP* protein family [36]. The conserved protein motif identifies motif1 and motif5 as the characteristic motifs of *Brassica juncea* var. *tumida*. This finding suggests that these genes have been relatively conserved during the evolution of this species.

The results of cis-acting elements present in the promoter of the members of the *Brassica juncea* var. *tumida PEBP* gene family showed that each gene contains various promoter cis-acting elements such as GARE-motif, p-box, and AuxRR-core for hormone regulation; AE-box and LAMP-element for light response; MBS and TC-rich for stress response; and HD-zip and CAT-box for growth and development. *MFT*-like genes integrate ABA and GA signaling pathways to control seed germination [42]. ABRE elements respond to GA; GARE-motif, TATC-box, and p-box respond to ABA. The results of cis-acting element analysis revealed that all *BjMFT* genes contain ABRE and p-box, which is consistent with the result of a previous study [30]. The light-responsive elements were mainly present in the *MFT*-like subfamily, with a higher distribution in the *FT*-like and *ybhB*-like subfamilies. The *FT*-like subfamily regulates plant flowering mainly under photoperiodic conditions. The growth and development–related elements are mainly present in the *TFL1*-like subfamily, which also reflects the primary function of this family in maintaining the nutritional growth of plants and the infinite growth state of inflorescences. All these elements have their specific functions and are involved in the regulation of gene expression. These elements are involved in the transcriptional regulation of genes via their binding with regulatory proteins and are thus important for the analysis of possible signaling pathways as well as functions. Therefore, the members of the *PEBP* gene family may play diverse functions during the growth and development of *Brassica juncea* var. *tumida*.

The specific expression patterns of genes in tissues usually reflect their biological functions. The RNA-Seq data obtained from different tissues of *Brassica juncea* var. *tumida* showed that the expression of both *BjTFL1* and *BjTFL3* was detected in YA1–YA4; these genes were highly expressed at the stage of stem inflation and thereafter, implying that these two genes are involved in the inflation or growth and development of *Brassica juncea* var. *tumida*. *BjATC2*, *BjATC3*, *BjBFT1*, *BjBFT2*, and *BjTFL2* genes showed a weak increase in expression in the YA3 period. YA3 is the period of stem inflation and the transition from nutritional to reproductive growth in *Brassica juncea* var. *tumida*. In *Arabidopsis* sp., the *AtATC*, *AtBFT*, and *AtTFL* genes repress flowering [17]. Whether the *BjATC2*, *BjATC3*, *BjBFT1*, *BjBFT2*, and *BjTFL2* genes in *Brassica juncea* var. *tumida* have similar functions warrant further studies. Owing to the high similarity of homologous gene sequences on the same branch in molecular evolutionary tree, primers do not distinguish between *BjPEBP* homologs. In *Arabidopsis* sp., *TSF* overexpression results in significantly early flowering [43]. In *Brassica juncea* var. *tumida*, *BjTSF* was expressed in the leaf, flower, and fruit pod; the expression was particularly high in the fruit pod. Therefore, *TSF* regulates plant flowering and probably seed development. *BjBFT1* was detected in all tissues but showed relatively high expression in the root, stem, and leaf. This finding is consistent with that of a previous study by Zhang et al. [36] who stated that *BFT* expression is relatively high in the stem and leaf of *Prunus yedoensis* and *Rosaceae occidentalis*. In *Arabidopsis* sp., *MFT4* plays a redundant role in flowering [9]. *BjMFT4* expression was noted to be higher in the root, stem, and leaf than in the flower and fruit pod, suggesting that *BjMFT4* is involved in nutritional growth, but not reproductive growth, in *Brassica juncea* var. *tumida*. *BjTFL1* belongs to the *TFL1*-like subfamily. Members of the *TFL1*-like subfamily aid in flower-forming transformation and inhibit flowering [41]. The expression of *BjTFL1* was higher in the leaf and flower than in other tissues. *BjPYBHB1*, a homolog of *PYBHB*, was highly expressed in the flowers of *Brassica juncea* var. *tumida*. However, to the best of our knowledge, no function of *PYBHB* has been reported yet. *BjFT* and *BjTSF* belong to the *FT-*like subfamily. They promote the flowering of plants. This result suggests that *PEBP* genes play an important role in different stages of the growth and development of *Brassica juncea* var. *tumida*.

Through subcellular localization prediction, *BjFT1* was observed to localize at multiple sites; experimental validation revealed that it localizes primarily on the plasma membranes. Plant 14-3-3 proteins are involved in the flowering, growth, and developmental processes [44]. Most proteins that interact with 14-3-3 proteins contain the following motifs; RSXpSXP [45], RXSXpSXP [46], RXF/YpSXP [47], and YpTV [48]. The multiple sequence alignment result showed that Bj*PEBP* proteins contain the RXF motif. Yeast two-hybrid experiment showed that Bj14-3-3 protein interacted with BjFT1 protein, suggesting that BjFT1 protein has a similar function with FT in *Arabidopsis* that regulate the flowering and seeding process of *Brassica juncea* var. *tumida*.

To the best of our knowledge, this study was the first to identify 21 *BjPEBP* genes in *Brassica juncea* var. *tumida* and reveal the roles of these genes in plant growth and development. This study speculated that these genes are involved in various processes such as hormone response, flowering transition of plants from nutritional to reproductive growth, and morphological structural changes. Our results may provide a reference for further studies on the molecular mechanism of the *BjPEBP* gene family of *Brassica juncea* var. *tumida* as well as a theoretical basis for molecular breeding.

## Conclusions

A genome-wide analysis was performed in this study, which resulted in the identification of a total of 21 *BjPEBP* genes of *Brassica juncea* var. *tumida*. Based on the classification of *PEBP* genes in *Arabidopsis* sp., these 21 genes were categorized into four subfamilies: *FT-*like, *MFT*-like, *TFAL1*-like, and *ybhB*-like. Of these 21 *BjPEBP* genes, 20 were located on 11 chromosomes and the remaining one was anchored in a contig. Based on the results of motif analysis, it appears that the *BjPEBP* genes are highly conserved. Although some genes show high expression during the growth and development of *Brassica juncea* var. *tumida*, the expression of some other genes is low.

## Materials and methods

### Plant materials and growth conditions

*Brassica juncea* var. *tumida* cultivar *Yonganxiaoye* was provided by Dr. Jinjuan Shen of the Institute of Chongqing Fuling Agricultural Sciences and used to analyze gene expression patterns. Seeds were sowed into nutrient soil and cultured at a constant temperature of 22 °C in long-day photoperiod (16 h of light, eight hours of dark) in the culture room.

### Identification of *PEBP* proteins in *Brassica juncea* var*. tumida*

The genome data of *Brassica juncea* var. *tumida*, *Brassica napus L.* (Bna_zs11) and *Brassica nigra* (Bnigra_N100.v2) were downloaded from the Brassica Database (BRAD; http://brassicadb.cn/) [2, 49]. *Arabidopsis PEBP* gene data were obtained from the TAIR database (https://www.arabidopsis.org/download/index-auto.jsp?dir=/download_files/Proteins). The Hidden Markov Model of the *PEBP* gene (PF01161) was downloaded from the Pfam website (http://pfam.xfam.org/family/PF01161). The PF01161 was searched in all protein sequences of *Brassica juncea* var. *tumida*, *Brassica napus L.* and *Brassica nigra* using the Hmmer software with an E-value of < 1.2e-12, and the screened out results were submitted to Pfam, NCBI CDD, and SMART for further verification [50–52].

### Sequence and molecular evolutionary analysis

The ClustalW program was used to perform multiple alignments of *PEBP* protein sequences from *Brassica juncea* var. *tumida*, *Arabidopsis*. A phylogenetic tree was constructed using MEGA 10.2.6 software [53] and the NJ method based on the passion correction model and bootstrap test replication 1000 times [54]. A gene structure diagram was drawn using the online software of the GSDS 2.0 server [55]. The physical location data of *BjPEBP* genes were retrieved from *Brassica juncea* var. *tumida*. Conserved protein motifs were identified using default parameters for the Multiple Em for Motif Elicitation (MEME) website (https://memesuite.org/meme/doc/meme.html?man_type=web), and a maximum of ten motifs were sat. The subcellular location of *BjPEBPs* was PSORT website (https://wolfpsort.hgc.jp). Using Expasy analysis, the physicochemical properties of *BjPEBP* gene family proteins. Finally, 1500-bp the 5’ sequence was used as each *PEBP* gene’s promotor region to analyze the cis-acting elements using PlantCARE (http://bioinformatics.psb.ugent.be/webtools/plantcare/html/) [56].

### Expression profile of *PEBP* genes

RNA-sequencing (RNA-seq) data were downloaded from the NCBI Sequence Read Archive database. The accession numbers are, SRX108496 (Dayejie [DY] stems, a mutant variety without inflated stems, were collected 22 weeks after seeding), SRX108498 (YA1; Yonganxiaoye [YA] stems were collected 18 weeks after seeding), SRX108499 (YA2; YA stems were collected 20 weeks after seeding), SRX108500 (YA3; YA stems were collected 22 weeks after seeding), SRX108501 (YA4; YA stems were collected 25 weeks after seeding), and SRX108502 (YAr; YA mix roots were collected 20 and 22 weeks after seeding). The computed reads per kilobase of transcript per million (RPKM) value was referred to in our previous report [57]. Screening of *PEBP* family genes data from raw data and using TBtools with selecting log scale, horizontal clustering, and the rest of the parameters are default to analyze the gene expression level.

### RNA extraction and real-time quantitative PCR analysis

Root, stem, leaf, flower, and fruit pod’s tissues were collected. Then, total RNA was extracted from different plant materials using RNA Plant Kit (Takara, Qingdao, China), and then reverse transcription was conducted using the PrimeScript™ 1st Strand cDNA Synthesis Kit (Takara, Qingdao, China) to get genome DNA. Real-time quantitative reverse transcription-polymerase chain reaction (qRT-PCR) was performed with 20-µL volume using SYBR qPCR Master Mix (Vazyme, Nanjin, China). The internal reference gene for qRT–PCR was *Bj18s*; Table S1 lists gene-specific primers.

Three replicate samples of each period were subjected to three biological replicates using the BioRad IQ5 Real-Time PCR instrument (BioRad Laboratories, Hercules, CA, USA). Amplification parameters were as follows: activation at 50 °C for two minutes, predenaturation at 95 °C for two minutes, denaturation at 95 °C for 15 s, and annealing at 60 °C for one minute for 40 cycles. Finally, the relative gene expression level was calculated using the 2^−ΔΔCt^ method [58].

### Subcellular localization of BjFT1 protein

The *BjFT1* gene was cloned into PCAMBIA1300-35 S-GFP vector and transformed into Agrobacterium tumefaciens strain LBA4404. Primers were designed according to the sequences of the *BjFT1* gene (Table S1). Agrobacterium containing only the *GFP* reporter gene and *Agrobacterium* containing the *BjFT1* gene was injected into *Nicotiana benthamiana* leaves, respectively. The transient transgenic *Nicotiana benthamiana* were darkened for 24 h and incubated under normal conditions for three days, and protein localization was observed under fluorescence microscopy.

### Yeast two-hybrid experiment

Total leaf RNA was extracted from *Brassica juncea* var. *tumida* and reverse transcribed to obtain cDNA. Primers were designed according to the sequences of *BjFT1* and *Bj14-3-3* genes (Table S1), and PCR amplified the target genes. Restriction endonucleases, *EcoRI* and *BamHI*, cut the pGADT7 and pGBKT7 vectors, ligating target genes to construct a recombinant vector. The Plasmids of pGBKT7-BjFT1 + pGADT7-Bj14-3-3, pGADT7 + pGBKT7-BjFT1, pGBKT7 + pGADT7-Bj14-3-3, pGADT7-T + pGBKT7-53, and pGADT7 + pGBKT7 combinations of transformed yeast receptor cells were coated onto two-deficiency SD medium and incubated upside down at 30 °C for 2–3 days. Colonies larger than 2 mm in diameter were transferred to a four-deficiency SD medium and incubated upside down at 30 °C for 4–5 days.

## Supplementary Information

**Additional file 1.**

## Data Availability

The following information was supplied regarding data availability: Data is available at NCBI SRA: SRX108496, SRX108498–SRX108502.

## Abbreviations

PEBP: Phosphatidylethanolamine-binding protein
FT: FLOWERING LOCUS T
TSF: TWIN SISITER OF FT
TFL1: TERMINAL FLOWER 1
BFT: BROTHER OF FT AND TFL1
MFT: MOTHER OF FT AND TFL1
ATC: ARABIDOPSIS THALLANA CENTRORADIALIS
CO: CONSTANS
SAM: shoot apical meristem
SOC1: SUPPRESSOR OF OVEREXPRESSION OF CONSTANS1
FUL: FRUITFUL
AP1: APETALA1
LFY: LEAFY
NCBI: National Center for Biotechnology Information
CDD: Conserved domain database
aa: amino acid
PI: isoelectric point
Mb: millions of bases
NJ: neighbor-joining
ABRE: abscisic acid response element
qRT-PCR: Quantitative real-time PCR
GSDS: Gene Structure Display Server
BRAD: Brassica Database
MEME: Multiple Em for Motif Elicitation
RNA-seq: RNA-sequencing
RPKM: reads per kilobase of transcript per million
ABA: abscisic acid
GA: gibberellin acid
