# Supplementary material for: Genome-wide analysis and identification of the PEBP genes of Brassica juncea var. Tumida
Source: BMC Genomics. 2022 Jul 23;23:535. doi: 10.1186/s12864-022-08767-3 (PMC9308242; doi:10.1186/s12864-022-08767-3)
Supplement: Supplementary file 1 — Additional file 1. [file 12864_2022_8767_MOESM1_ESM.docx]

**Supplementary information**

**Additional file 1:**

**Table S1. List of** primer sequences.

| Primer name | Prime sequence |
| --- | --- |
| qBjTFL1-F | CAACGGCCATGAGCTTTTCC |
| qBjTFL1-R | GCTAGGCCTTGGCAACTCAT |
| qBjTFL6-F | GGTTGGGATTCACCGGTACA |
| qBjTFL6-R | TGAAGCTGGCTCTTTCTGGG |
| qBjFT1-F | GCCAAGAGTTGAGATTGGTGGAG |
| qBjFT1-R | ACCAGTGAAGATATTCTCGGAGGTG |
| qBjMFT4-F | AACGATCACAAGATCCCGGG |
| qBjMFT4-R | TGTCAAAACCGCTTCTCCGA |
| qBjATC1-F | CGGCCAAACATAGGGATCCA |
| qBjATC1-R | GGTCTCACGCTGACAGTTGA |
| qBjTSF1-F | ACCTGCCACAACTGGAACAA |
| qBjTSF1-R | CTCAGCGAACTCACGAGTGT |
| qBjBFT1-F | GTTGAAATTGGCGGCCATGA |
| qBjBFT1-R | GTCTGAACAGCACAAACGCA |
| Bj18s-F | TCGTAGTTGGACTTAGGGTGGG |
| Bj18s-R | CAAATGCTTTCGCAGTTGTTCG |
| 1300-BjFT1-F | gagctcggtacccggggatccATGTCTTTAAGTAATAGAGATCCTCTTGTGG |
| 1300-BjFT1-R | gcccttgctcaccatgtcgacCTAGCTTCTTCGACCTCCGCA |
| BD-BjFT-F | atggccatggaggccgaattcATGTCTTTAAGTAATAGAGATCCTCTTGTGG |
| BD-BjFT-R | ccgctgcaggtcgacggatccCTAGCTTCTTCGACCTCCGCA |
| AD-Bj14-3-3-F | gccatggaggccagtgaattcATGTCATCATCAGGATCCGACAA |
| AD-Bj14-3-3-R | cagctcgagctcgatggatccTCAGTTCTCAGTGGCWTCMG |
